# Variable Virulence Factors in *Burkholderia pseudomallei* (Melioidosis) Associated with Human Disease

**DOI:** 10.1371/journal.pone.0091682

**Published:** 2014-03-11

**Authors:** Derek S. Sarovich, Erin P. Price, Jessica R. Webb, Linda M. Ward, Marcos Y. Voutsinos, Apichai Tuanyok, Mark Mayo, Mirjam Kaestli, Bart J. Currie

**Affiliations:** 1 Global and Tropical Health Division, Menzies School of Health Research, Darwin, Northern Territory, Australia; 2 Department of Tropical Medicine, Medical Microbiology and Pharmacology, University of Hawai’i, Honolulu, Hawai’i, United States of America; 3 Infectious Diseases Department, Royal Darwin Hospital, Darwin, Northern Territory, Australia; Tulane University School of Medicine, United States of America

## Abstract

*Burkholderia pseudomallei* is a Gram-negative environmental bacterium that causes melioidosis, a potentially life-threatening infectious disease affecting mammals, including humans. Melioidosis symptoms are both protean and diverse, ranging from mild, localized skin infections to more severe and often fatal presentations including pneumonia, septic shock with multiple internal abscesses and occasionally neurological involvement. Several ubiquitous virulence determinants in *B. pseudomallei* have already been discovered. However, the molecular basis for differential pathogenesis has, until now, remained elusive. Using clinical data from 556 Australian melioidosis cases spanning more than 20 years, we identified a *Burkholderia mallei-*like actin polymerization *bimA*_Bm_ gene that is strongly associated with neurological disease. We also report that a filamentous hemagglutinin gene, *fhaB*3, is associated with positive blood cultures but is negatively correlated with localized skin lesions without sepsis. We show, for the first time, that variably present virulence factors play an important role in the pathogenesis of melioidosis. Collectively, our study provides a framework for assessing other non-ubiquitous bacterial virulence factors and their association with disease, such as candidate loci identified from large-scale microbial genome-wide association studies.

## Introduction

*Burkholderia pseudomallei* and its equine-adapted clonal derivative *Burkholderia mallei* (the causal agent of glanders) are formidable pathogens. In 2012, *B. pseudomallei* and *B. mallei* were upgraded to Tier 1 Select Agent status in the United States, a ranking reserved for only those infectious agents that pose the greatest risk to human and animal health and which also includes *Bacillus anthracis* (anthrax) and *Yersinia pestis* (plague) [1]. Our knowledge of the global distribution of *B. pseudomallei* is expanding as regions of endemicity continue to be unveiled, with this bacterium now considered endemic in tropical northern Australia, many countries in Asia, selected locations in the Americas and Africa, and several islands in the Indian and Pacific Ocean islands [2]. Northeast Thailand and the tropical Top End of the Northern Territory in Australia are hyperendemic for this bacterium, with melioidosis being a major cause of community-acquired pneumonia and sepsis in these regions [3], [4].

The fulminating septicemic form of melioidosis has a mortality rate up to 90%, and overall mortality in northeastern Thailand remains at 40% despite over two decades of research and improved therapy [4]. In contrast, the mortality rate of *B. pseudomallei* infection in northern Australia is now comparatively low at 10–15% [3]. In addition, up to 5% of Australian melioidosis cases have a neurological presentation with an encephalomyelitis syndrome that rarely occurs in Thailand or elsewhere in Southeast Asia [3], [5], [6]. Despite the clear importance of *B. pseudomallei* and the disease it causes, the role of genetic variation in the *B. pseudomallei* genome and its association with disease severity has remained elusive.

Several ubiquitous virulence factors in *B. pseudomallei* have previously been identified, including the cytotoxin Burkholderia lethal factor 1 [7], capsular polysaccharide I [8], the cluster I type VI secretion system [9], and the Bsa type III secretion system cluster 3 [10]. However, the wide spectrum of melioidosis presentations has led to speculation that variably present virulence factors may influence disease severity. To examine this potential, we identified two variably present virulence candidates, determined their prevalence among 556 Australian *B. pseudomallei* strains of clinical origin collected over a 24-year period, and finally, compared their association with disease presentation and severity.

## Results and Discussion

The first variable virulence candidate we examined was *Burkholderia* intracellular motility factor A (BimA), a putative type V effector protein [11] required for actin-based motility. BimA subverts host actin dynamics, enabling bacterial movement between eukaryotic cells and evasion of immune surveillance [11], [12]. All *B. pseudomallei* strains encode for BimA*;* however, approximately 12% of northern Australian strains possess a *bimA* variant that shares 95% homology with *Burkholderia mallei* (*bimA*_Bm_) [13], [14]. *B. mallei* is an equine-adapted clone of *B. pseudomallei* that causes glanders, a highly infectious disease primarily of horses but which is often fatal in humans if untreated [15]. BimA_Bm_ shares only 54% homology with BimA_Bp_, although both variants impart actin polymerization capabilities [11]. The BimA_Bp_ and BimA_Bm_ carboxy-terminal ends share homology with the *Yersinia* autosecreted adhesion YadA [16], a critical virulence factor in *Y. enterocolitica* that enables host cell invasion while resisting immune attack [17].

Using multivariate logistic regression models to control for all potential confounders, we found a significant association between *bimA*_Bm_ and neurological melioidosis (*p*<0.001), with patients infected by the *bimA*_Bm_ variant being 14 times more likely to present with neurological involvement compared with patients infected with the *bimA*_Bp_ variant (95% CI = 4.7–44.6). No covariates were found to be associated with neurological disease, indicating that *bimA*_Bm_ was the only variable predictive of neurological presentation. In contrast, patients infected with *B. pseudomallei* possessing the *bimA*_Bp_ variant were twice as likely to develop pneumonia compared with those infected with the *bimA*_Bm_ variant (*p* = 0.028; 95% CI = 1.1–3.5), although several covariates existed for this presentation; specifically, being indigenous Australian Aboriginal, having chronic lung or heart disease, hazardous alcohol use, being older, not having a malignancy, and no history of kava use. Pneumonia is the most common melioidosis presentation in Australia, occurring in 51% of cases [3]; thus, the identification of multiple covariates for pneumonia is not unexpected. The *bimA*_Bm_ variant has not yet been observed in clinical and environmental isolates from Thailand or in other endemic regions of Southeast Asia including Cambodia, Laos and Vietnam [14], although the *bimA*_Bm_ variant has been found in two isolates originating from India [18]. Thus, the apparent absence of the *bimA*_Bm_ variant in Southeast Asia could explain the reduced incidence of encephalomyelitis observed in this region.

Previous studies in a murine model have demonstrated that intranasal delivery of *B. pseudomallei* or *B. mallei* can result in invasive entry into the central nervous system, probably via the olfactory nerve, enabling the bacteria to directly infect the brain without hematogenous spread [19], [20]. In addition, neurological degeneration has been reported in a study on experimental glanders in horses [21]. Based on these studies, the prevailing hypothesis is that an intranasal route of infection substantially increases the risk of bacterial migration to the brain. We demonstrate that *B. pseudomallei* strains possessing the *bimA*_Bm_ variant have a substantially heightened ability to cause central nervous system disease, possibly due to enhanced bacterial motility along the olfactory and other nerve pathways [20]. Although it remains unclear to what extent an inhalational/intranasal route of infection contributes to neurological melioidosis in humans, our findings suggest that inhalation or nasal aspiration of a *bimA*_Bm_ variant substantially increases the likelihood of developing a brain infection compared with a *bimA*_Bp_ variant.

The second variable virulence candidate we tested encodes for a filamentous hemagglutinin (FhaB). *B. pseudomallei* contains one ubiquitous FhaB (encoded by *BPSS1727*) [22] and three variably present FhaB loci. Of the latter, *B. pseudomallei* strains can contain one, two or all three *fhaB* genes [23]. These variably present genes are located on genomic islands (GIs), and previous reports have suggested a role in *B. pseudomallei* virulence [23], [24]. Of note, the variably present FhaB3 (encoded by *BPSS2053* in *B. pseudomallei* K96243) is an anti-macrophage factor [25] involved in host epithelial cell attachment [24], the formation of multinucleated giant cells and actin-associated membrane protrusions [26]. *BPSS2053* shares 47% homology with *Bordetella pertussis* FhaB, an adhesin that facilitates attachment to the host cell during infection, the secretion of which is dependent upon FhaC [27]. Due to its immunogenicity, FhaB from *B. pertussis* is as an essential component of the acellular pertussis (whooping cough) vaccine [28].

The majority (83%) of *B. pseudomallei* isolates examined in our study possessed *fhaB*3. Using multivariate logistic regression models to control for all potential confounders, we found that patients harboring *fhaB*3-positive *B. pseudomallei* were twice as likely to be blood culture-positive (*p* = 0.028; 95% CI = 1.1–3.4), although there was no significant association with septic shock or mortality. Several covariates existed for blood culture-positivity; namely, being indigenous Australian Aboriginal, being older, having diabetes, immunosuppression or malignancy, and hazardous alcohol use. In contrast, we observed a strong correlation between *fhaB*3 absence and cutaneous melioidosis without sepsis, with *fhaB*3*-*negative *B. pseudomallei* being four times more likely to be the infecting organism in these patients (*p* = 0.001; 95% CI = 1.8–8.1). Cutaneous melioidosis without sepsis occurs as the primary presentation in 13% of Australian cases [3], mostly in those with no identified risk factors and commonly in children; furthermore, fatalities have yet to be documented in this cohort [29].

It is intriguing that *fhaB*3 has, to date, been found in 100% of Thai *B. pseudomallei* strains [23] compared with only 83% of Australian strains, and as such it is tempting to speculate that *fhaB3* may contribute to increased mortality in Thai melioidosis patients. However, our study did not find an association between *fhaB3* and mortality. In addition, the potential contribution of *fhaB*3 to differential mortality rates between Thailand and Australia requires evaluation in the context of differences in access to diagnostic and therapeutic resources, and in particular, to state-of-the-art critical care management of severe sepsis [30]. Although further work is needed to identify other virulence determinants associated with mortality, it is striking that *B. pseudomallei* strains lacking *fhaB*3 are significantly less likely to cause severe disease. Indeed, it is possible that *fhaB*3, in concert with another virulence factor, contributes to adverse melioidosis outcomes.

In addition to the *fhaB*3 and *bimA* genes, we screened our isolate collection for the *Burkholderia thailandensis*-like flagellum and chemotaxis (BTFC) and the *Yersinia*-like fimbrial (YLF) gene clusters, which are mutually exclusive in the *B. pseudomallei* genome [31]. The BTFC and YLF gene clusters are putative virulence determinants, with the YLF cluster previously shown to be over-represented in clinical isolates [31]. Like *fha*B3, the YLF cluster is more common in Thailand than Australia and as expected, we found that the majority of isolates in our study possessed the BTFC cluster (79%). We performed BTFC/YLF analysis across 543 strains from our clinical collection with the same parameters used for *bimA* and *fhaB*3 to assess correlation with disease presentation and severity metrics. Interestingly, no statistically significant correlations for clinical presentations or disease severity metrics were identified for BTFC or YLF (Table S3). There was, however, an overrepresentation of the YLF gene cluster in strains from remote regions of Australia. The lack of correlation of any clinical presentation or disease severity metric with BTFC/YLF in our Australian isolates is itself significant as it highlights the nonrandom nature of our *bimA* and *fhaB3* findings.

Associations between variable virulence factors and adverse disease outcome are still poorly understood, although salient examples have been documented in well-studied human pathogens, most notably nontyphoid *Salmonella* [32] and Shiga-toxin producing *Escherichia coli* [33]. Our study has conclusively identified, for the first time, variably present *B. pseudomallei* virulence factors that are associated with specific melioidosis presentations. The *bimA*_Bm_ variant was shown to be highly predictive of neurological melioidosis, and most likely explains why this disease presentation is more frequently observed in Australia than Thailand. Further, we have demonstrated that *B. pseudomallei* strains lacking *fhaB*3 are significantly associated with non-severe cutaneous disease compared with strains containing *fhaB*3, a finding which potentially contributes to the lower mortality rate of melioidosis in Australia. In contrast, strains containing *fhaB3* were associated with positive blood cultures, but not septic shock or mortality, suggesting that additional virulence factors remain to be identified. In contrast, we did not find any association between BTFC/YLF and disease presentation or disease severity. Collectively, our findings represent a fundamental step towards understanding the molecular basis for variable disease presentation caused by this high-threat biological agent, and ongoing microbial genome-wide association studies are likely to uncover additional virulence signatures.

## Materials and Methods

### Ethics Statement

Our study examined primary *B. pseudomallei* isolates obtained from 556 melioidosis patients from the Darwin Prospective Melioidosis Study, which commenced at Royal Darwin Hospital, Northern Territory, Australia in October 1989. Ethics approval for this study was obtained through the Human Research Ethics Committee of the Northern Territory Department of Health and Menzies School of Health Research, approval number HREC 02/38 (Clinical and Epidemiological Features of Melioidosis) with written informed consent obtained from patients. All patient data were de-identified prior to analysis.

### PCR Assay Design and PCR Conditions

The presence of *fhaB*3, *bimA*_Bm_, *bimA*_Bp_, BTFC and YLF among the *B. pseudomallei* isolates was determined using real-time PCR. For *bimA*_Bm_, 0.9 µM of each primer (AGCGCTTCGCGCATCTAC, CGCGTTAAACGCCGTACTTTC) (Invitrogen, Mulgrave, VIC, Australia) and 0.25 µM TaqMan minor groove-binding probe (6FAM-ATGCGCTCGCTATC) (Applied Biosystems, Foster City, CA, USA) was used to generate a 104 bp amplicon within this 579 bp gene (corresponding to *BURPS668_A2118* in *B. pseudomallei* MSHR668). Identical primer (GGAAGCTTTGGCGTGCATAT, CCCATGCCTTCCTCGACTAAT) and probe (6FAM-ACGTCACTGCCAATC) concentrations were used to detect a unique 60 bp fragment within the 1.5 kb *bimA*_Bp_ locus (encoded by *BPSS1492* in *B. pseudomallei* K96243 [34]). For *fhaB*3 detection, 0.3 µM each primer (GACGCGGCACGTCTGATC, CGCGGATAAAACTCGGATTG) and 0.2 µM probe (6FAM-AACCAGGTCAACAGCA) was used to generate a conserved 58 bp product within the 9.3 kb *fhaB*3 gene (*BPSS2053* in K96243). For BTFC and YLF detection, primers and probes were designed targeting *lafU* or *BPSS0124* (corresponding to BTFC and YLF loci, respectively). BTFC and YLF detection was multiplexed into a single assay containing 0.3 µM of each primer (TGTTTCGCAGCGAGGATGTC, CCCACCGTCAAGCCGATT) and 0.2 µM of probe (6FAM-CGTCCGACCGAGTGC) targeting BTFC and identical concentrations of primers (GTGCCTGCAACGCTAATCG, CGCACTGATAGCCGGAATAGAG) and probe (VIC-ACTGGATTTACTCAGCGCAT) targeting YLF. Positive controls for *bimA*_Bp_, *bimA*_Bm_, BTFC, YLF *or fhaB*3 were included on each PCR plate to ensure accurate assay performance. All PCRs used TaqMan Universal Mastermix (Applied Biosystems) and were carried out to a final volume of 5 µL, in duplicate. PCR instrumentation and conditions were as previously described [35].

### Clinical and Epidemiological Features of Melioidosis Cases

We categorized the 556 patients into six primary (and mutually exclusive) diagnoses: pneumonia, genitourinary involvement, blood culture positivity but no identifiable focus, localized skin infection without sepsis, neurological melioidosis and internal soft tissue abscess. Disease severity metrics were also included, where relevant: blood culture-positive growth of *B. pseudomallei*, septic shock, and mortality. Melioidosis risk factors were scored as either present or absent for the following, as previously defined for the Darwin Prospective Melioidosis Study [3]: chronic lung disease, chronic renal disease, diabetes, hazardous alcohol use, heart disease (defined as either rheumatic heart disease or congestive cardiac failure), immunosuppression, kava use and malignancy. Criteria for defining hazardous alcohol use, chronic lung disease, chronic renal disease and septic shock have previously been detailed [3]. Last, four epidemiological factors were included to rule out potential confounding influences: gender, ethnicity (either indigenous Australian Aboriginal or not), age, and probable acquisition of infection locality. Locality was divided into three regions: urban Darwin, rural Darwin, or elsewhere in Australia, including the remote Top End of the Northern Territory.

### Statistical Analysis

All statistical analyses were carried out using Stata version 13.0 (StataCorp LP, College Station, TX, USA). Bivariate analysis was performed using the Pearson’s χ^2^ test where expected frequencies were >5 in all cells, or the two-sided Fisher’s exact test where expected frequencies were <5 in one cell (Tables S1 and S2). The Wilcoxon rank-sum test was used to compare age of diagnosis with *bimA* or *fhaB3* status. To rule out confounding factors (e.g. age, geographic location, or risk factors) associated with *bimA* or *fhaB*3 status and clinical outcome, multivariate logistic regression analyses were performed using a stepwise backwards-elimination procedure as previously described [3]. We used a threshold of *p = *0.1 for inclusion of covariates in the logistic regression model, followed by a *p* = 0.01 for primary associations and a *p = *0.05 for secondary associations.

## Supporting Information

Table S1 (DOCX)

Table S2 (DOCX)

Table S3 (DOCX)
