# Supplementary material for: Variable Virulence Factors in Burkholderia pseudomallei (Melioidosis) Associated with Human Disease
Source: PLoS One. 2014 Mar 11;9(3):e91682. doi: 10.1371/journal.pone.0091682 (PMC3950250; doi:10.1371/journal.pone.0091682)
Supplement: Table S1 — (DOCX) [file pone.0091682.s001.docx]

**Supplemental information: Virulence determinants of melioidosis (Sarovich *et al.*)**

Table S1. Bivariate clinical associations with *bimA*

| **Primary diagnosis** | ***bimA*_Bm_^a^** | ***bimA*_Bp_^a^** | ***p*** |
| --- | --- | --- | --- |
| Pneumonia | 25 (36%) | 264 (54%) | 0.005 |
| Genitourinary presentation | 9 (13%) | 63 (13%) | >0.1 |
| Blood culture positive, no focus | 9 (13%) | 60 (12%) | >0.1 |
| Localized skin infection without sepsis | 7 (10%) | 67 (14%) | >0.1 |
| Neurological presentation | 9 (13%) | 5 (1%) | <0.001 |
| Soft tissue abscess | 3 (4%) | 11 (2%) | >0.1 |
| **Disease severity metrics** |  |  |  |
| Blood culture positive | 39 (57%) | 280 (58%) | >0.1 |
| Septic shock | 15 (22%) | 113 (23%) | >0.1 |
| Died from infection | 11 (16%) | 74 (15%) | >0.1 |
| **Risk factors** |  |  |  |
| Hazardous alcohol use | 26 (38%) | 188 (39%) | >0.1 |
| Diabetic | 22 (32%) | 211 (43%) | 0.071 |
| Renal disease | 1 (1%) | 66 (14%) | 0.004 |
| Kava use | 13 (19%) | 10 (2%) | <0.001 |
| Malignancy | 3 (4%) | 36 (7%) | >0.1 |
| Rheumatic heart disease/congestive cardiac failure | 7 (10%) | 37 (8%) | >0.1 |
| Chronic lung disease | 17 (25%) | 124 (26%) | >0.1 |
| Immunosuppression | 2 (3%) | 42 (9%) | 0.099 |
| No risk factors | 14 (20%) | 94 (19%) | >0.1 |
| Darwin, NT urban | 11 (16%) | 257 (53%) | <0.001 |
| Darwin, NT rural | 3 (4%) | 67 (14%) |  |
| Remote/not NT | 55 (80%) | 163 (34%) |  |
| Gender (male) | 44 (64%) | 318 (65%) | >0.1 |
| Indigenous Australian | 48 (70%) | 243 (50%) | 0.002 |
| Median age (years) | 42 | 50 | 0.005 |

**^a^**Percentages indicate the proportion of cases positive for a given primary diagnosis, disease severity metric or risk factor according to *bimA*_Bm_ or *bimA*_Bp_.

NB. *n=*556 for all tests. The exception is blood culture, in which *n=*548.

Abbreviations: *bimA*, Burkholderia intracellular motility factor A gene; NT, Northern Territory, Australia; N/A. not applicable.
