# Supplementary material for: Variable Virulence Factors in Burkholderia pseudomallei (Melioidosis) Associated with Human Disease
Source: PLoS One. 2014 Mar 11;9(3):e91682. doi: 10.1371/journal.pone.0091682 (PMC3950250; doi:10.1371/journal.pone.0091682)
Supplement: Table S2 — (DOCX) [file pone.0091682.s002.docx]

Table S2. Bivariate clinical associations with *fhaB*3

| **Primary diagnosis** | ***fhaB*3 -ve^a^** | ***fhaB*3 +ve^a^** | ***P*^b^** |
| --- | --- | --- | --- |
| Pneumonia | 34 (47%) | 255 (53%) | >0.1 |
| Genitourinary presentation | 7 (10%) | 65 (13%) | >0.1 |
| Blood culture positive, no focus | 5 (7%) | 64 (13%) | >0.1 |
| Localized skin infection without sepsis | 21 (29%) | 53 (11%) | <0.001 |
| Neurological presentation | 3 (4%) | 11 (2%) | (>0.1) |
| Soft tissue abscess | 1 (1%) | 13 (3%) | (>0.1) |
| **Disease severity metrics** |  |  |  |
| Blood culture positive | 34 (48%) | 285 (60%) | 0.059 |
| Septic shock | 11 (15%) | 117 (24%) | 0.094 |
| Died from infection | 9 (13%) | 76 (16%) | >0.1 |
| **Risk factors** |  |  |  |
| Hazardous alcohol use | 20 (28%) | 194 (40%) | 0.045 |
| Diabetic | 26 (36%) | 207 (43%) | >0.1 |
| Renal disease | 11 (15%) | 56 (12%) | >0.1 |
| Kava use | 0 (0%) | 23 (5%) | (0.059) |
| Malignancy | 7 (10%) | 32 (7%) | >0.1 |
| Rheumatic heart disease/congestive cardiac failure | 9 (13%) | 35 (7%) | >0.1 |
| Chronic lung disease | 14 (19%) | 127 (26%) | >0.1 |
| Immunosuppression | 12 (17%) | 32 (7%) | (0.008) |
| No risk factors | 20 (28%) | 88 (18%) | 0.055 |
| Darwin, NT urban | 55 (76%) | 213 (44%) | <0.001 |
| Darwin, NT rural | 3 (4%) | 67 (14%) |  |
| Remote/not NT | 14 (19%) | 204 (42%) |  |
| Gender (male) | 46 (64%) | 316 (65%) | >0.1 |
| Indigenous Australian | 34 (47%) | 257 (53%) | >0.1 |
| Median age (years) | 48 | 50 | >0.1 |

**^a^**Percentages indicate the proportion of cases positive for a given primary diagnosis, disease severity metric or risk factor according to *fhaB3* presence or absence.

***^b^****p* values in parentheses were determined using the Fisher’s exact test due to one expected value being <5.

NB. *n=*556 for all tests. The exception is blood culture, in which *n=*548.

Abbreviations: *fhaB*3, filamentous hemagglutinin 3 gene; NT, Northern Territory, Australia; N/A, not applicable.
