# Supplementary material for: Variable Virulence Factors in Burkholderia pseudomallei (Melioidosis) Associated with Human Disease
Source: PLoS One. 2014 Mar 11;9(3):e91682. doi: 10.1371/journal.pone.0091682 (PMC3950250; doi:10.1371/journal.pone.0091682)
Supplement: Table S3 — (DOCX) [file pone.0091682.s003.docx]

Table S3. Bivariate clinical associations with BTFC/YLF

| **Primary diagnosis** | **BTFC^a^** | **YLF^a^** | ***P*** |
| --- | --- | --- | --- |
| Pneumonia | 226 (53%) | 58 (50%) | >0.1 |
| Genitourinary presentation | 52 (12%) | 18 (16%) | >0.1 |
| Blood culture positive, no focus | 54 (13%) | 14 (12%) | >0.1 |
| Localized skin infection without sepsis | 56 (13%) | 14 (12%) | >0.1 |
| Neurological presentation | 3 (3%) | 11 (3%) | >0.1 |
| Soft tissue abscess | 10 (2%) | 3 (3%) | >0.1 |
| **Disease severity metrics** |  |  |  |
| Blood culture positive | 248 (59%) | 65 (58%) | >0.1 |
| Septic shock | 97 (23%) | 28 (24%) | >0.1 |
| Died from infection | 64 (15%) | 18 (16%) | >0.1 |
| **Risk factors** |  |  |  |
| Hazardous alcohol use | 166 (39%) | 46 (40%) | >0.1 |
| Diabetic | 182 (43%) | 47 (41%) | >0.1 |
| Renal disease | 52 (12%) | 15 (13%) | >0.1 |
| Kava use | 13 (3%) | 10 (9%) | 0.007 |
| Malignancy | 32 (7%) | 7 (6%) | >0.1 |
| Rheumatic heart disease/congestive cardiac failure | 32 (7%) | 9 (8%) | >0.1 |
| Chronic lung disease | 113 (26%) | 26 (23%) | >0.1 |
| Immunosuppression | 33 (8%) | 11 (10%) | >0.1 |
| No risk factors | 80 (19%) | 23 (20%) | >0.1 |
| Darwin, NT urban | 215 (50%) | 47 (41%) | 0.001 |
| Darwin, NT rural | 59 (14%) | 6 (5%) |  |
| Remote/not NT | 154 (36%) | 62 (54%) |  |
| Gender (male) | 46 (64%) | 316 (65%) | >0.1 |
| Indigenous Australian | 34 (47%) | 257 (53%) | >0.1 |
| Median age (years) | 50 | 48 | >0.1 |

**^a^**Percentages indicate the proportion of cases positive for a given primary diagnosis, disease severity metric or risk factor according to BTFC or YLF.

NB. *n=*543 for all tests. The exception is blood culture, in which *n=*535.

Abbreviations: BTFC, *B. thailandensis*-like flagellum and chemotaxis cluster; YLF, *Yersinia*-like fimbrial cluster; NT, Northern Territory, Australia; N/A, not applicable.
